# Qualitative insights into how men with low-risk prostate cancer choosing active surveillance negotiate stress and uncertainty

**DOI:** 10.1186/s12894-017-0225-3

**Published:** 2017-05-08

**Authors:** Emily M. Mader, Hsin H. Li, Kathleen D. Lyons, Christopher P. Morley, Margaret K. Formica, Scott D. Perrapato, Brian H. Irwin, John D. Seigne, Elias S. Hyams, Terry Mosher, Mark T. Hegel, Telisa M. Stewart

**Affiliations:** 1 Department of Family Medicine, SUNY Upstate Medical University, 475 Irving Ave., Suite 200, Syracuse, NY 13210 USA; 2 Department of Public Health and Preventive Medicine, SUNY Upstate Medical University, 766 Irving Ave., Rm. 2262, Syracuse, NY 13210 USA; 3 Department of Psychiatry and Behavioral Sciences, SUNY Upstate Medical University, 750 E Adams St., Syracuse, NY 13210 USA; 4 Department of Psychiatry, Geisel School of Medicine at Dartmouth, Dartmouth-Hitchcock Medical Center, 1 Medical Center Dr., Lebanon, NH 03756 USA; 5 Division of Urology, Department of Surgery, University of Vermont College of Medicine, Fletcher House 301, 111 Colchester Ave., Burlington, VT 05401 USA; 6 Urology Section, Geisel School of Medicine at Dartmouth College, Dartmouth-Hitchcock Medical Center, 1 Medical Center Dr., Lebanon, NH 03756 USA; 7 Cancer Control Program, Norris Cotton Cancer Center, Dartmouth-Hitchcock Medical Center, 1 Medical Center Dr., Lebanon, NH 03756 USA

**Keywords:** Active surveillance, Prostatic neoplasm, Qualitative research, Coping behavior

## Abstract

**Background:**

Active surveillance is a management strategy for men diagnosed with early-stage, low-risk prostate cancer in which their cancer is monitored and treatment is delayed. This study investigated the primary coping mechanisms for men following the active surveillance treatment plan, with a specific focus on how these men interact with their social network as they negotiate the stress and uncertainty of their diagnosis and treatment approach.

**Methods:**

Thematic analysis of semi-structured interviews at two academic institutions located in the northeastern US. Participants include 15 men diagnosed with low-risk prostate cancer following active surveillance.

**Results:**

The decision to follow active surveillance reflects the desire to avoid potentially life-altering side effects associated with active treatment options. Men on active surveillance cope with their prostate cancer diagnosis by both maintaining a sense of control over their daily lives, as well as relying on the support provided them by their social networks and the medical community. Social networks support men on active surveillance by encouraging lifestyle changes and serving as a resource to discuss and ease cancer-related stress.

**Conclusions:**

Support systems for men with low-risk prostate cancer do not always interface directly with the medical community. Spousal and social support play important roles in helping men understand and accept their prostate cancer diagnosis and chosen care plan. It may be beneficial to highlight the role of social support in interventions targeting the psychosocial health of men on active surveillance.

## Background

The introduction of the prostate-specific antigen (PSA) blood test has increased detection of prostate cancer in the US, and most men are now diagnosed with localized, early stage and low-risk tumors (PSA < 10 ng/mL, clinical staging T1-T2a, and Gleason score ≤6) [1–3]. Expectant management strategies, including active surveillance (AS), are now being increasingly recommended to low-risk prostate cancer patients in an effort to avoid unnecessary treatment and its associated risks [1].

AS is a process of closely monitoring men with low-risk prostate cancer through PSA blood tests, digital rectal exams (DREs), ultrasounds, and prostate biopsies, with the goal of averting active treatment unless disease progression is detected or the patient chooses treatment [4, 5]. The small volume of research evaluating the psychosocial health and coping mechanisms of men following AS indicates that a lack of social support, illness uncertainty and anxiety are predictive factors in reduced quality of life among men with low-risk prostate cancer, and the psychosocial burden of living with prostate cancer affects adherence to AS and disease outcomes [6–8]. In addition, a recent study found that men who have low levels of coping confidence and high levels of treatment concern had higher intrusive thoughts about their cancer [9], underscoring the value of a better understanding of the coping strategies employed by men on active surveillance. Our research presented in this article investigates the primary coping mechanisms for men who have chosen to follow AS, with a specific focus on how these men interact with their social network as they negotiate the stress and uncertainty of their diagnosis and treatment approach.

## Methods

This project is an extension of a prior qualitative study grounded in self-regulation theory investigating the decision-making process for low-risk prostate cancer patients [10, 11]; the initial data collection procedures are described in detail elsewhere [12]. Briefly, patient and provider semi-structured interviews were conducted at two academic institutions in the northeastern US. Patient inclusion criteria was based on patient age of 18 years or older, diagnosis of T1 or T2 prostate cancer within the past year, PSA value ≤10, Gleason Score ≤ 6, adequate fluency in English, and consent to participate. Interviews were conducted via telephone at the participants’ convenience, and were transcribed by professional transcriptionists; transcriptions were proofread by an interviewer for assurance of quality and accuracy. The study was reviewed and approved by the institutional review boards (IRB) at Geisel School of Medicine at Dartmouth College, University of Vermont, and SUNY Upstate Medical University.

In the current study, only those transcripts from interviews with patients following AS were analyzed, as we were particularly focused on the coping and uncertainty management strategies of men who had chosen this treatment approach. Transcript data were analyzed following an immersion/crystallization process. Immersion and crystallization is a cyclical process of organizing and connecting data involving repeated readings of the data (immersion) followed by periods of reflection wherein themes and patterns are developed (crystallization); this process continues until meaningful patterns emerge from the data that can be well articulated and substantiated [13, 14]. Following this process, two authors (EMM and HHL) independently conducted initial coding of the transcripts, followed by a joint review and consolidation of the code list. Finalized codes and identified themes were then reviewed by the wider research team for confirmation. All transcript analysis was managed using ATLAS.ti, software version 7.

## Results

Fifteen men following AS were interviewed; all men were white, non-Hispanic, with a mean age of 65 years. The majority of the men interviewed were married (73.3%), with a college or graduate degree (66.7%), and were evenly split between working full time or retired. Details on respondent demographics can be found in Table 1.

**Table 1:** Summary of patient demographic characteristics

Patient characteristic	Mean (SD)
Age	65 (6.45)
	Count (%)
Race/Ethnicity	
White	15 (100)
Non-Hispanic	15 (100)
Marital Status	
Never Married	3 (20)
Married	11 (73.3)
Divorced	1 (6.7)
Employment	
Full Time	7 (46.7)
Retired	7 (46.7)
Declined to answer	1 (6.7)
Annual Income	
Less than $40,000	3 (20)
$40,000 or more	9 (60)
Declined to answer	3 (20)
Education	
High school graduate/GED^a^	2 (13.3)
Some college/technical school	2 (13.3)
College graduate	7 (46.7)
Graduate degree	3 (20)
Declined to answer	1 (6.7)
Household	
Living with spouse	11 (73.3)
Living alone	4 (26.7)
Insurance	
Private	7 (46.7)
Medicare	1 (6.7)
Medicaid	1 (6.7)
Medicare + Private	6 (40)

^a^ *GED* general educational development test

**Fig. 1:**
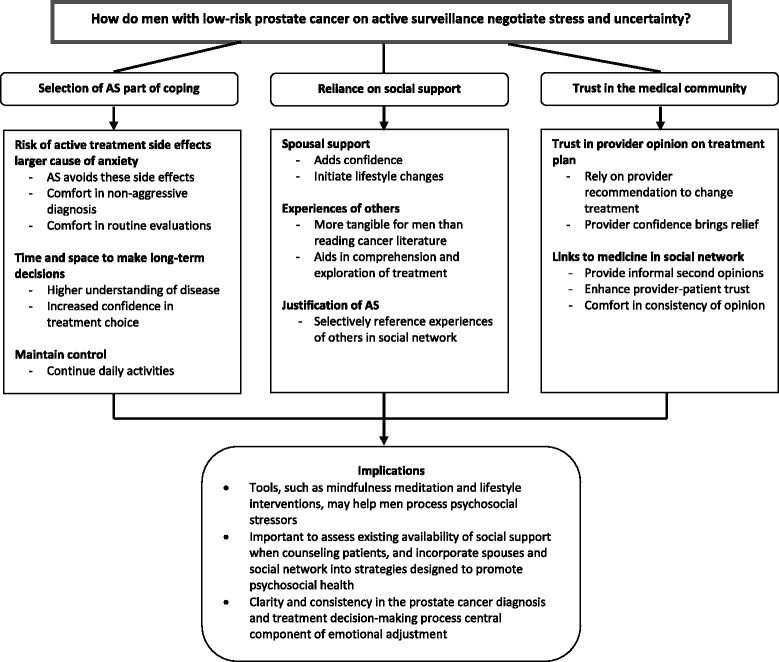
Negotiating Stress and Uncertainty in Active Surveillance

Three overarching themes emerged from our analysis of the interview transcripts. First, the choice to follow AS is related to how men cope with the anxieties experienced over the diagnosis and treatment of prostate cancer. Second, the reliance men place on individuals in their social networks during the decision-making process extends to the period following the decision, in which they adjust and learn to cope with their untreated cancer. Lastly, the trust men place in their providers and the medical community has a strong impact on their ability to follow AS while effectively managing uncertainty and anxiety. Figure 1 provides an overview of these themes.

### Selection of AS as part of coping

Ten participants described the risks associated with active treatment options as an area of significant concern. These men perceived urinary incontinence and erectile dysfunction as concrete and imminent risks, which would have a larger impact on their day-to-day lives than their current prostate cancer diagnosis. The potential of experiencing these side effects generated concern for several of the individuals interviewed. Additionally, several men discussed the negative impact erectile dysfunction would have on the health of their personal relationships with spouses and partners:

“I certainly let him know that my wife and I, we’re very close and active sexually, and very much in love with each other. So, he went through all of the potential outcomes of the surgery, none of which are very enticing to me.” [pt 13]

Participants viewed AS as a viable treatment option that would appropriately manage their cancer while avoiding the risk of negative side effects. Several men also expressed relief in the diagnosis of a non-aggressive cancer, stating that since their cancer was “controllable and treatable” they were able to take a “less emotional attitude” about treatment choices. These participants were reassured by the ability to follow routine evaluations to track the progression of their cancer:

“We’re going to deal with it in that I’m gonna have another biopsy this fall because it’ll be a year. That’s the way the active surveillance is set up…I just, I’d rather do it this way. I’d rather be goin’ up there and checkin’ in and havin’ a blood test than goin’ to the hospital for radiation or do this. If it’s not necessary, it’s a waste of money and time.” [pt 02]

It is important to note that the choice to follow AS did not eliminate all anxieties regarding the cancer diagnosis. Two participants felt increased worry in the period leading up to their next PSA test or biopsy. However, it appears that the anxiety felt prior to these regular checkups was less than that experienced when contemplating life with side effects from active treatment, and several men indicated feeling a sense of relief and reassurance after receiving benign test results:

“So, it’s seems like we are doing the right thing, and it gives me a little more peace of mind each three months when I go back in, well, we’re on the right track here.”[pt 01]

Some participants reported that following the AS plan allowed them valuable time to contemplate the need for future prostate cancer treatment, and what their preferred treatment choice would be. This contemplation period allowed men to reach a higher level of understanding of their disease, as well as a higher degree of confidence in their ability to make the right decision for themselves:

“In one way, it’s a relief because it can postpone any potential confrontation with, ‘Okay, doggone it. Now I’ve really got to make a decision for surgery or ignore the damn thing.’ So it’s been comforting.” [pt 13]

Several men also reported that following the AS treatment plan allowed them to continue living their lives as normal. These individuals valued this capability, and felt that many of their daily activities would face interference through more active treatment interventions:

“I had thought that the radiation treatment would be like you go like a couple of times, and you don’t. You go many times, almost every day for, like, a long time.” [pt 15]

The ability to continue routine activities allowed them to maintain a sense of control in a scenario with a large degree of uncertainty, and this control over daily life decreased the anxiety some men experienced over their diagnosis:

“They tell you, ‘You have prostate cancer,’ but, at the same time, ‘We’re monitoring it,’ and I’m kinda going on like, just, ah, normal living. And so I don’t - I don’t think about it that much.” [pt 03]

Comments from three men also suggest that the diagnosis of prostate cancer sparked a desire to “enjoy the rest of” their lives, and following AS was viewed as a window in time to be healthy and functional without treatment regimens and side effects.

### Reliance on social support

Ten of the men on AS expressed a strong sense of reliance on their spouses not only during the treatment decision-making process, but also during the period thereafter in which they adjusted to life on AS. Spouses were viewed as partners in this process, and their support brought added confidence to many of the participants:

“We say it’s a joint effort – we are in this together. And, you know, figure out what is the best decision for us.” [pt 02]

“But ya gotta have a good outlook. And I’ve got probably one of the most positive women that I’ve ever met in my life for a wife, which is a big help.” [pt 02]

Several participants mentioned that their spouses initiated lifestyle changes that they felt could potentially reduce the impact or slow the progression of prostate cancer. Similar to the maintenance of routine activities, these changes in habits were a way in which men could maintain a sense of control over and cope with their diagnosis:

“She packs my lunch in the morning and makes sure I have all the right stuff, and a lot of fruits and vegetables, and none of the sweets I loved having (*laughing*) …went on with the tea, only have minimal beef from the beef industry and a lot of fruits and vegetables, and trying to get an alkaline diet rather than an acidic diet. So that’s what I tried to do to counteract this.” [pt 01]

Participant responses also indicate that talking to and reflecting upon the experiences of individuals who had previously been diagnosed with prostate cancer is an important component of the decision to go on and stay on AS. Several participants described a process wherein after reviewing the medical literature offered by physicians, they sought out individuals within their social networks who had previous encounters with prostate cancer to learn about their experiences with treatment:

“Well, I’ve contacted several people, family and different relationships. Why you, you learn from them who has had it, and yes, I have contacted probably, oh, probably half a dozen different individuals.” [pt 01]

The majority of participants spoke with men who had undergone active treatment for prostate cancer, with only three speaking with individuals following AS. Most men spoke with family members and friends, but one individual specifically attended a prostate cancer support group meeting. Communication within the social network seemed to provide more tangible information to the participants compared to information they learned through reviewing medical literature; hearing about others’ experiences and opinions allowed the participants to contextualize the potential risks and outcomes of active treatment to their own lives.

Several participants referenced stories shared by friends or family members who had negative outcomes from active treatment as evidence to support their choice to follow AS:

“I was gonna say one of the factors that weighed a little into my decision was my friend who had this, and, you know, he felt like overreacted after. He said, ‘You know I just want to get rid of it,’ and like a year later he told me he regretted that decision, you know, having it removed. He should have waited – that maybe because he’s having a lot of side effects, I don’t know.” [pt 05]

Additionally, two participants referenced stories from family members who also chose not to seek active treatment for cancer; these family members lived longer than expected and died from causes other than cancer:

“And my father had prostate cancer. His, they claimed, had spread and were really pretty pessimistic. But he was older. And he decided not to do anything. And he lived – I don’t know – something like seven or eight years and died of a heart attack.” [pt 07]

By referencing these stories from their social networks, the participants were able to justify their own choice to follow AS by providing evidence that a) a slow-moving cancer would not be the ultimate cause of death, and b) active treatment would have a larger, negative impact on quality of life than the prostate cancer itself.

### Trust in the medical community

Six men directly referenced conversations with friends or family members who were linked to the medical community, mostly through occupation. The advice received from these individuals was particularly emphasized, as their connection to the medical community added weight and legitimacy to their opinions over that of other members of the participants’ social networks. For some men, the advice gained from these individuals was viewed as an informal second opinion:

“Being a professor…and having a number of students who were – they’re both MD students and PhD students – I talked to them about it and…the basic conclusion that I got from them was I would be insane to go through radiation treatment with a Gleason index, you know, so extremely low, with just one sample.” [pt 15]

Furthermore, the decision to follow AS was strengthened for participants when the second opinions obtained from specialists, or the opinions they heard from their friends or family members within the medical community, were concordant with the recommendations received by their providers. This consistency increased the trust participants held with their own providers, and brought a sense of relief and confidence that they made an appropriate decision in following AS:

“I had been doing some processing and it was reassuring to hear him say the same thing as the first doctor.” [pt V01]

Seven participants also mentioned trust in their provider as they discussed their rationalization of the choice to follow AS. Several men took comfort in the notion that their providers would be able to recognize changes in their disease and make responsive recommendations to change treatment plans if or when necessary:

“I put a lot of faith in them. I have a lot of trust in them. They’re very good at what they’re doing.” [pt 9]

“I really would wait for the physician to say, you know, it’s time to take it out, take the prostate out.” [pt 06]

## Discussion

Much of the approach that men on AS take to negotiating the anxiety and uncertainty of their low-risk prostate cancer stems from the identification of their cancer as an indolent – or not life-threatening – disease with an extended time line of progression. Several studies evaluating the treatment decision-making process for men with low-risk prostate cancer generally conclude that men choosing aggressive interventions, such as surgery or radiation, do so from a strong desire to eradicate or cure a disease they view as life threatening, especially if they have a longer life expectancy at the age of diagnosis; conversely, men choosing AS tend to not view their prostate cancer as an immediate health threat [15–20]. The first theme developed in our analysis builds on this understanding of why men choose AS: the men in our sample did not view their prostate cancer as a direct threat to their well-being, but rather considered the potential side effects of aggressive treatment as threats. In this manner, the anxiety men on AS experience can stem in great part from prostate cancer treatment. The first approach to combat treatment-related anxiety for the individuals in our sample was to avoid the negative outcomes and risks associated with active treatment, and following AS was an agreeable method by which they could do this while ensuring their disease was not neglected.

The statements made by men in our sample also highlight the importance of maintaining control in coping with the unease of untreated cancer. For some men within our sample, this control took the form of maintaining routine activities without interruption, while for others it took the form of altering dietary and exercise habits. If men on AS characterize their prostate cancer as a slow-moving, non-threatening disease, any interruption to daily life would indicate that the cancer is not indolent and trigger a re-assessment of their illness representation. Additionally, adopting changes in diet and exercise are tangible actions that men perceive as beneficial to their disease state, and bring a sense of assurance to an otherwise uncertain scenario [21].

Concordant with prior investigations, our findings indicate that some men on AS may experience increased anxiety prior to routine follow-up procedures [6, 22]. Despite these initial anxieties, several of the men in our sample reported feeling reassured by the findings of their follow-up testing, which reaffirmed the suitability of AS. In fact, recent evidence indicates that routine testing plays an important role in the psychological health of men on AS by mollifying fears associated with disease progression. Thus, testing is an important component of anxiety management [22]. The time line of the AS protocol also afforded men the opportunity to become more familiar with all prostate cancer treatment options by extending the timeframe during which they could gather and digest information. Some men in our sample were concerned over the potential need to select an active treatment in the future due to disease progression. Following AS allowed these individuals the time necessary to reach a comfortable familiarity with available active treatment options and reduced their current anxiety over future changes. This finding complements that of qualitative research conducted by Volk et al. among men with localized prostate cancer, which concluded that the ability to slow the decision-making process allowed men on AS to gain a better understanding of the AS approach to cancer treatment and overcome the prevailing heuristic for immediate and aggressive treatment [19].

Existing literature focused on the role of social support during the diagnosis and decision-making process for prostate cancer treatment indicates that men seek opinions from their spouses and social connections as they make their treatment decisions [6, 17, 19, 23]. Comments from the men in our sample reflect this influence of social support, and particularly highlight the role of personal experiences with prostate cancer shared from within men’s social networks in the decision-making process. It appears that these anecdotal stories may be more informative or salient for men compared to information gathered from the general literature, or, at times, even from medical professionals.

The results of our study further indicate that this reliance on the social network does not end once the treatment decision has been made; rather, men continue to rely upon spousal support as they make adjustments to life with untreated cancer, and continue to justify their treatment choice by learning from and referencing the experiences of others. The intersection between feedback from social connections and feedback from the medical community is also a significant component of the confidence men have in their decision to follow AS. Concordance between the opinions obtained from men’s providers and those received from individuals within their social networks further legitimized AS as a valid treatment option for men in our sample, especially when those social connections had ties to the medical community.

### Clinical implications

Providers may not always be aware of the psychosocial issues men with low-risk prostate cancer on AS experience. However, there are tools or potential interventions providers can share with their patients to help them process psychosocial stressors associated with their cancer diagnosis and management, including instruction in mindfulness meditation, cognitive-behavioral stress management, and diet and exercise lifestyle interventions [24–26]. A study of one such lifestyle intervention conducted in California, US, found that men felt the program supported feelings of optimism, hope and well-being [27]. Several men in our sample reported making similar lifestyle changes independently, indicating that the topics addressed in these interventions are salient among AS patients and present a viable avenue through which their psychosocial health can be supported.

Spouses and family members often act as health advocates, sources of emotional support, and facilitate patient-provider communication among men with prostate cancer [28–30]. Spouses of prostate cancer patients can frequently experience issues related to emotional wellness, balancing their health needs with those of the patient, and lack of communication [28]. Therefore, it may be important for providers to offer support resources to men’s spouses and families as well. Engaging spouses and families in support services not only addresses their individual anxieties related to the prostate cancer diagnosis, but educate these individuals on AS management and enable them to adopt supportive roles for the prostate cancer patient [31].

Clarity in the diagnosis and treatment decision-making process is a central, salient component of emotional adjustment for men with low-risk prostate cancer [32]. Men who receive corroborating information from both their social network and from within the medical community may have less confusion and anxiety over their treatment decision, and increased trust in provider recommendations [33]. It is important for providers to acknowledge the role outside opinion – whether gathered internally or externally from the medical community – has on men’s perceptions of prostate cancer.

### Limitations

This study was conducted with a small convenience sample at academic institutions with multidisciplinary cancer centers. The multidisciplinary centers allow patients to receive timely feedback from providers in multiple specialties, often in the same day, prior to making treatment decisions. This study site likely influenced the degree to which the experiences of the men in our sample are generalizable to those of men receiving treatment outside a multidisciplinary setting. Additionally, the participants in our study were largely homogenous in demographic characteristics, and our findings may not generalize to men from other demographic groups and settings.

Some men in our sample may have been following AS for longer periods than others. It is possible that men with more months on AS were able to use that time to gain a more in-depth understanding of their disease and security in their treatment decision compared to other men in the sample. Additionally, the variability in time since treatment decision among men in our sample may have introduced recall bias in the content of the interviews. Future investigations targeting specific subgroups of prostate cancer patients may yield insight into variations in coping strategies among men based on time since diagnosis.

Finally, the research question described in this report emerged from a prior analysis originally targeting the decision-making process for men diagnosed with low-risk prostate cancer. As such, the interview transcripts analyzed in this project were originally constructed to elicit responses regarding that subject area, rather than directly focusing on coping and the role of men’s social networks in managing stress and anxiety.

## Conclusions

The decision to follow AS in many ways reflects the need to maintain control, as this treatment choice avoids more aggressive treatment options. Men on AS manage their prostate cancer-related anxiety by both maintaining a sense of control over their daily lives, as well as relying on the support provided them by their social networks and the medical community. Social networks, and more specifically spouses, support men on AS by encouraging lifestyle changes and serving as a resource to discuss and ease cancer-related stress. Men who are able to maintain a sense of trust and connection to their providers experience less worry and anxiety over their untreated prostate cancer.

It is important to recognize that these coping mechanisms or approaches to life on AS are not independent from one another, but rather collectively influence the patient’s perception of his illness. Education and support services offered to patients should incorporate their friends and family members. Furthermore, physicians may be better prepared to address areas of confusion, contradiction and anxiety for their patients on AS by identifying the availability of social support and acknowledging the feedback obtained from within their patients’ social networks regarding prostate cancer.

## Abbreviations

AS: Active surveillance
DRE: Digital rectal exam
IRB: Institutional review board
PSA: Prostate-specific antigen
US: United States
